# A dual-task-embedded virtual reality system for intelligent quantitative assessment of cognitive processing speed

**DOI:** 10.3389/fnhum.2023.1158650

**Published:** 2023-03-30

**Authors:** Yuzhao Zhou, Yixuan Zhao, Zirui Xiang, Zhixin Yan, Lin Shu, Xiangmin Xu, Lulu Zhang, Xiang Tian

**Affiliations:** ^1^School of Electronic and Information Engineering, South China University of Technology, Guangzhou, China; ^2^School of Future Technology, South China University of Technology, Guangzhou, China; ^3^Pazhou Lab, Guangzhou, China; ^4^Zhongshan Institute of Modern Industrial Technology of South China, University of Technology, Zhongshan, China; ^5^Department of Psychiatry, Guangzhou First People’s Hospital, The Second Affiliated Hospital, South China University of Technology, Guangzhou, China

**Keywords:** cognitive processing speed, evaluation, virtual reality, dual-task, behavior data, machine learning

## Abstract

**Introduction:**

Processing speed is defined as the ability to quickly process information, which is generally considered as one of the affected cognitive functions of multiple sclerosis and schizophrenia. Paper–pencil type tests are traditionally used in the assessment of processing speed. However, these tests generally need to be conducted under the guidance of clinicians in a specific environment, which limits their application in cognitive assessment or training in daily life. Therefore, this paper proposed an intelligent evaluation method of processing speed to assist clinicians in diagnosis.

**Methods:**

We created an immersive virtual street embedded with Stroop task (VR-Street). The behavior and performance information was obtained by performing the dual-task of street-crossing and Stroop, and a 50-participant dataset was established with the label of standard scale. Utilizing Pearson correlation coefficient to find the relationship between the dual-task features and the cognitive test results, and an intelligent evaluation model was developed using machine learning.

**Results:**

Statistical analysis showed that all Stroop task features were correlated with cognitive test results, and some behavior features also showed correlation. The estimated results showed that the proposed method can estimate the processing speed score with an adequate accuracy (mean absolute error of 0.800, relative accuracy of 0.916 and correlation coefficient of 0.804). The combination of Stroop features and behavior features showed better performance than single task features.

**Discussion:**

The results of this work indicates that the dual-task design in this study better mobilizes participants’ attention and cognitive resources, and more fully reflects participants’ cognitive processing speed. The proposed method provides a new opportunity for accurate quantitative evaluation of cognitive function through virtual reality.

## 1. Introduction

Processing speed is a indicator of brain efficiency, which represents how fast an individual can perform various cognitive operations, and is closely related to the ability to perform higher-order cognitive tasks (Lichtenberger and Kaufman, 2012; Ojeda et al., 2012). Processing speed is now one of the key diagnostic items in the new diagnostic framework defined in the Diagnosis and Statistical Manual 5*^th^* edition (DSM-5) (Boland, and Robert, 2015). The reduction of processing speed is generally considered as one of the important signs of mild cognitive impairment (MCI), multiple sclerosis (MS) and schizophrenia (Ferreira, 2010; Knowles et al., 2010; Kern et al., 2011; Lahera et al., 2017; Lu et al., 2017).

Stroop test are often used to assess selective attention and processing speed (Stroop et al., 1935). The classic Stroop test has three different tasks: (a) color naming: name the color of ink patches, (b) word reading: read words that are the names of colors, (c) color-word interference: name the color ink in which incongruent color words are printed. When the meaning of a word is different from the color of the ink, personal attention will be distracted and decisions will be interfered, resulting in more time to react. This is the Stroop effect, performance on this task is a measure of cognitive inhibition or selective attention. Both the color naming and word reading performance are considered measures of processing speed (Jensen, 1965; Lezak et al., 2004). Some studies have shown that patients with impaired processing speed perform significantly worse in Stroop tasks (Macniven et al., 2008; Bodling et al., 2009; Denney and Lynch, 2009; Sisco et al., 2016). The traditional Stroop test is paper-pencil type. However, due to less interference and one-to-one guidance of professionals, typical test environments inevitably reduce the similarity with daily environmental needs, reduce the ecological effectiveness, and limit the application of this test in cognitive assessment and training in real life (Chaytor and Schmitter-Edgecombe, 2003).

The development of virtual reality (VR) provides a new method for cognitive assessment; it can build a realistic virtual environment (VE), simulate real life activities, and provide users with immersive experience (Rus-Calafell et al., 2018). Due to the advantages of VR in replicating daily life, the cognitive assessment method of VR is more ecologically effective than the paper-pencil test (Lalonde et al., 2013). At present, a large number of studies have applied VR to the evaluation and rehabilitation of diseases (Han et al., 2012; Shin et al., 2015), VR has gradually broken through the laboratory into clinical practice. A lot of research has verified the effectiveness of transferring Stroop testing to VR environment (Parsons et al., 2011, 2013; Lalonde et al., 2013; Parsons and Barnett, 2018), and reveal that VR can provide an ecological perspective for the actual cognitive function in daily life, and can be seen as a supplement to the traditional assessment test of complex cognitive ability.

With the rapid growth of artificial intelligence technology, an increasing amount of research has applied machine learning to the diagnosis of cognitive diseases (Bratic et al., 2018; Salmanpour et al., 2019; Carvalho et al., 2020). Disease assessment is a complex process, and data sources are diverse, such as medical imaging, psychological testing, etc. Machine learning has the ability to extract useful information from complex and large data sets and integrate multimodal data, which helps to develop more objective and accurate disease diagnosis models. Until recently, some researches have tried to combine VR with machine learning (Alcañiz Raya et al., 2020; Yeh et al., 2020; Tsai et al., 2021). Users’ activities in VE can provide researchers not only task performance data, but also behavioral data, which can be used as digital biomarkers to assess cognitive decline and, to some extent, reflect user behavior in the real world (Mandryk and Birk, 2019). The combination of machine learning and virtual reality may bring new and promising methods for the evaluation of nervous system diseases.

This research developed VR-Street by building an immersive virtual street scene, and embedded Stroop tasks in it as a distraction task for street-crossing. When subjects participate in tasks in VR, we collected their behavior and task performance data, and analyzed them through machine learning to estimate processing speed scores. Herein, we describe (1) the design of immersive virtual environment and dual-task experiment of virtual reality street; (2) data collection and analysis methods; (3) the evaluation model based on machine learning and the experiment results. Finally, we discussed the future direction of improvement.

## 2. Related work

The advantage of VR is that it can make participants feel “present” and provide individuals with stimulation close to the reproduction of daily life. Street-crossing is a common daily activity. Safe crossing requires individuals to estimate vehicle arrival time in advance and to avoid vehicles while walking. Previous studies have shown that visual acuity, attention, and processing speed are important factors in predicting collisions in simulated street-crossing tasks (Dommes et al., 2013; Dommès et al., 2015). The application of VR provides a safe simulation environment for street-crossing. Some studies conducted individual behavior or cognitive rehabilitation research by simulating street-crossing in VR. Weiss et al. (2003) first proposed a virtual street to improve the effectiveness of cognitive intervention for patients with unilateral visual space neglect. Navarro et al. (2013) compared the performance of the healthy control group and stroke participants in the virtual street, and verified the availability of the virtual street in the field of cognitive rehabilitation. Wagner et al. (2019) analyzed the difficult factors that may affect cognitive rehabilitation training in virtual street, such as relevant lanes, traffic speed and gap size between vehicles. Another advantage of VR is that it is convenient to record the behavior data of participants. Stratton et al. (2017) conducted a clinical trial on 26 patients with multiple sclerosis and 19 healthy participants using a simulated street-crossing task. They collected behavioral variables such as waiting time, time of crossing the street, and times of turning heads. They found that compared with the control group, multiple sclerosis patients spent more time crossing the street and were closer to oncoming vehicles, which proved that it was feasible to distinguish multiple sclerosis patients using street crossing behavioral data.

In addition, the use of VR as a supplementary assessment tool can overcome concerns about the predictive value of pen-paper tests in daily life. Distraction and interference in daily life are lacking in the paper-pencil cognitive testing environment. VR has the ability to provide interference factors in typical living environment under controllable conditions. A lot of research has embedded Stroop test into virtual reality scenes, including classroom scenes, apartment scenes and driving scenes (Parsons et al., 2011, 2013; Lalonde et al., 2013; Parsons and Barnett, 2018). Parsons et al. (2011) reported that VR Stroop task was more sensitive to attention and inhibition than traditional tests. Dual-task is a way to assess cognitive ability in situations of distraction and interference. The dual-task experimental paradigm requires simultaneous attention and cognition, which are particularly important in daily activities that require simultaneous coordination of two or more tasks (Baddeley et al., 1997; Proctor and Read, 2009). According to the central capacity sharing model, when performing dual-tasks, the two tasks must share limited attention and processing capacity, resulting in a decline in performance. Neider et al. (2011) and Banducci et al. (2016) simulated the dual-task of using mobile phones when crossing the street in a virtual environment, proving that the distraction of dual-task will reduce the success rate of crossing and lead to longer preparation time. Chang et al. (2020) created a Stroop task embedded virtual reality driving system, requiring participants to complete the Stroop task while driving on the simulator to study the application of head mounted displays/flat screen displays in assessing and enhancing cognitive processing ability, and proved that the system is suitable for cognitive assessment.

Quantitative cognitive assessment results can help patients understand their cognitive status more specifically and conduct self-monitoring. Many studies have proposed methods to calculate cognitive assessment scores (Wild et al., 2008; Brouillette et al., 2013; Moore et al., 2017; Oliveira et al., 2018). For example, Chua et al. (2019) calculated the scores based on the participants’ performance in VR tasks, and conducted statistical analysis with the scores of traditional cognitive screening tools to evaluate the effectiveness. In recent years, machine learning technology has been gradually applied to the research of cognitive assessment. Unlike classified tasks, researchers use patients’ activity data, such as task performance data or behavior data, to train machine learning models, and map the results to specific scale scores. Dawadi et al. (2016) proposed a method for predicting residents’ clinical cognitive scores using activity behavior. Using a smart home to collect residents’ daily activity performance data, extracted relevant statistical features, and utilized machine learning to predict clinical scores. The predicted results obtained a statistically significant correlation (*r* = 0.72) with the clinical score. Jung et al. (2019) proposed a series of serious mobile games to assess cognitive function. The game-specific performance data of 12 stroke patients were collected and used to train the supervised machine learning model to estimate the MMSE scores. The result showed that they can estimate the MMSE scores with a normalized root mean square error of 5.75%. However, this approach is still rare in research related to virtual reality.

Although VR-Street has been applied in the above research, there are still some deficiencies. First of all, most studies investigated the application of a single virtual street-crossing task in cognitive assessment. However, in real life, people often need to face multiple cognitive tasks at the same time. For healthy participants, a single street-crossing task may not fully stimulate their cognitive ability. It is necessary to design a dual-task that combines different cognitive tasks to evaluate the processing speed of participants more comprehensively. Secondly, most studies focus on the classification of cognitive impairment, while quantitative assessment methods based on continuous scores are rarely considered. Although some studies have proposed different calculation methods for cognitive assessment scores, these methods are only for the software in the report, and cannot convert the specific score into the score of the clinical assessment tool, which results in the limitation of clinical application. Additionally, the number of data features used for analysis is small, and behavioral data such as trajectory or velocity are not considered, and the performance of multiple tasks is not considered. Regression methods are mainly based on statistical methods. Advanced computing methods, such as machine learning, are not applied to high-dimensional data processing. In order to solve the above problems, this research proposes a new VR-Street, which is deployed using a head-mounted display (HMD) to provide an immersive experience. The system includes a dual-task consisting of a street-crossing task and a Stroop task, in which the Stroop task is presented in a mixed form of audio and video. In the VR scene, the performance data of two tasks were measured at the same time, including the reaction time, accuracy rate in the Stroop task, and the behavior data such as the trajectory and the number of head turns in the street-crossing task. A total of 68 features were measured. Finally, an intelligent evaluation model was constructed using machine learning.

## 3. Materials and methods

### 3.1. Framework

**FIGURE 1:**
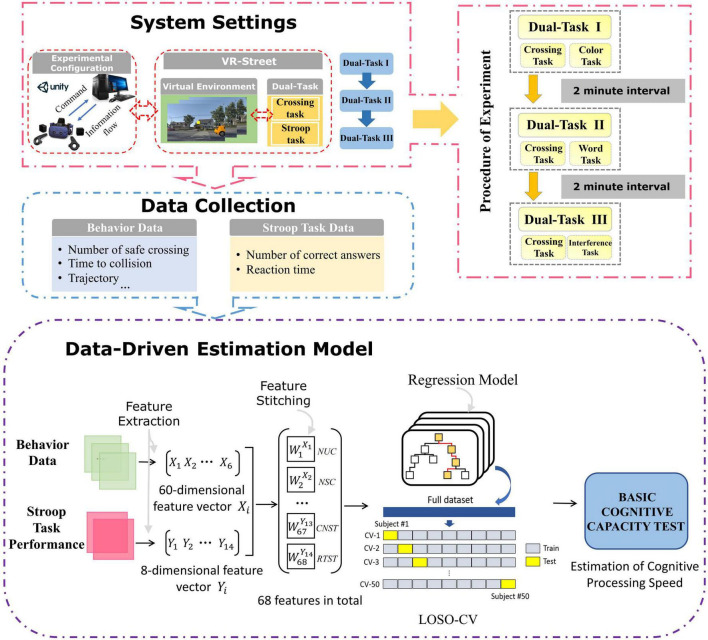
System framework.

Virtual scene was developed by the game engine Unity3D, mainly including a two-lane street and its surrounding environment. Dual-task was applied to VR scenes, including street-crossing task and Stroop task. Integrating the Stroop test into the VR street-crossing task may better approximate the complexity of real-life situations. When crossing the street, participants need to deal with the interference of Stroop test. This increases the difficulty of the task and requires participants to complete the task as soon as possible while maintaining their concentration. Participants wore a HMD to complete the whole task in the VE and conducted human-computer interactions through the VR controller. The system was divided into three dual-tasks, including Dual-Task I, Dual-Task II and Dual-Task III. Each dual-task was composed of street-crossing task and congruent or incongruent Stroop task. Dual-task performance was recorded in real time, including the behavior of street-crossing and Stroop task performance. Sixty-eight dimensional features were extracted for training a regression model to estimate standard neuropsychological test scores. Figure 1 describes the architecture of this research, including the experimental software and hardware configuration, the composition of virtual reality system, experimental process, data collection content and the process of training evaluation model.

### 3.2. Equipment

Virtual reality devices included HTC VIVE Pro HMD, emitters, and VIVE Controllers. VIVE controllers were used to interact with virtual scenes. Experiment was monitored by experimenters using desktop computers (Intel i7 processors, 8 GB of memory, NVIDIA GeForce GTX 1070 graphics cards). Computers and virtual reality devices were connected via data lines.

### 3.3. System design

**FIGURE 2:**
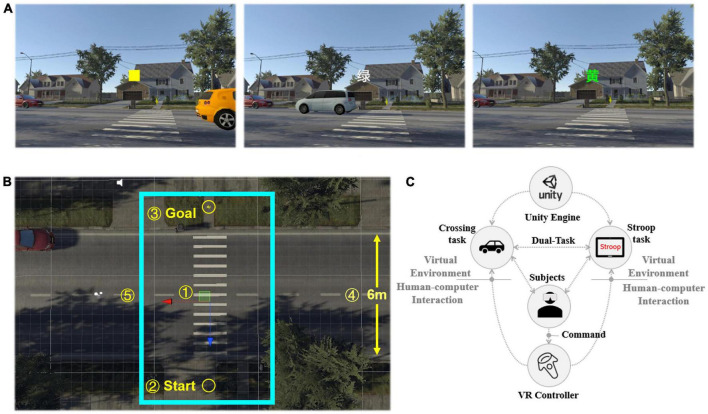
Virtual scene overview. **(A)** Three examples of virtual environment. Left: VE of Dual-Task I with a color-matching task. Middle: VE of Dual-Task II with a word-matching task. Right: VE of Dual-Task III with an interference-matching task. **(B)** Schematic structure of the street scene. is the zebra crossing; is the start point; is the destination for crossing the street; indicates the width of the street; shows the area where participants are allowed to move. The red arrow in the figure is the orientation of the *x*-axis, while the blue arrow is the orientation of the *y*-axis. **(C)** System design.

We built a street VE in Unity using the Suburb Neighborhood House Pack resource package. Figure 2A shows the screen capture of the virtual environment. It is a two lane highway with vehicles in two directions. In the VE, the number of lanes and the speed of vehicles are the main factors affecting the difficulty of crossing the road (Wagner et al., 2019). According to the common speed in the community, the speed of the vehicle is set to 30 km/h. To control the difficulty of crossing the street, we specifically design the vehicle gap so that 50% of the vehicle gap can allow participants to cross safely. The zebra crossing is set in the middle of the street, and participants need to cross the street on the zebra crossing. An arrow on the other side of the street indicates the participant’s destination. The total width of the lane is set to 6 m, extending 0.5 m on both sides of the lane to identify the safe distance between the participant’s starting point and end point and the lane. Figure 2B shows the schematic structure of the virtual street. The participant’s walking and stopping are controlled by the VR controller. The walking speed is fixed at 1.5 m/s, which is the normal human walking speed. During the experiment, the participant always performs the task from the first person perspective. The design overview is shown in Figure 2C.

**FIGURE 3:**
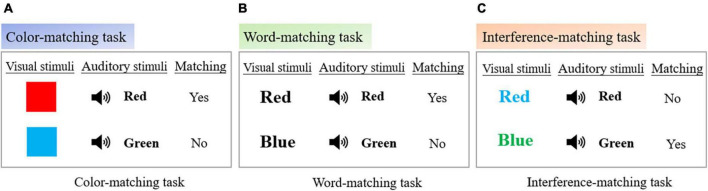
Stroop task settings. **(A)** Color-matching task settings. **(B)** Word-matching task settings. **(C)** Interference-matching task settings.

The Stroop task was embedded into the virtual environment. Traditional Stroop test includes three sub-tests, color-naming, word-reading and color-word interference. Each sub-test involves different cognitive control processes. To make a more comprehensive assessment of the participants’ cognitive control and processing speed, all the sub-tests were integrated into the VR scene. In this paper, Stroop task was transformed into matching task using visual and auditory stimuli, in which visual stimuli were colored blocks or Chinese characters in the screen, and auditory stimuli were color pronunciation in headphones. It has been proved that this method is as effective as the traditional Stroop (Parsons and Barnett, 2019). The specific settings of the three tasks are as follows:

• Color-matching task: Small blocks of different colors would appear in the middle of the HMD screen, and the pronunciation of the colored blocks would be output in the headset. The participants needed to judge whether the pronunciation matched the color of the block within the specified time (Figure 3A).

• Word-matching task: Chinese characters printed in white ink would appear in the middle of the HMD screen, and the pronunciation of the color of Chinese characters would be output in the headset. Participants needed to judge whether the pronunciation matched the color represented by Chinese characters within the specified time (Figure 3B).
• Interference-matching task: Chinese characters printed in color ink would appear in the middle of the HMD screen, and the pronunciation of the color of the Chinese characters would be output in the headset. Participants needed to judge whether the pronunciation matched the color of Chinese characters within the specified time (Figure 3C).

There were 72 stimuli in the color-matching task, word-matching task and interference-matching task, the presentation time of each visual stimulus was 2 seconds. Participants were required to complete their judgment within 2 seconds, and the next stimulus was updated if the judgment was still not completed at the end of the allotted time. The total time for each task was 144 s. When the judgment was correct, the headset would output a positive prompt tone, while when the judgment was wrong or the judgment was not completed after timeout, it would be a negative prompt tone.

### 3.4. Task settings

The system was divided into three dual-tasks: Dual-Task I, participants were required to complete both street-crossing task and color-matching task; Dual-Task II, participants were required to complete both street-crossing task and word-matching task; and Dual-Task III, participants were required to complete both street-crossing task and interference-matching task. The three dual-tasks were executed in turn, and the time of each dual-task was 144s. The crossing task required the participants to walk from the starting point through the zebra crossing to the position indicated by the arrow. In the process of crossing the street, the participants needed to turn left and right frequently, pay attention to the driving conditions of vehicles on the street, and choose an appropriate time to cross the street safely. While avoiding the vehicle, the participants also needed to judge the consistency of visual and auditory stimuli in the Stroop task. A safe crossing is when there are more than 1.5 s between leaving the respective roadway and the arrival of the car at this point (Simpson et al., 2003). Whether it was safe crossing or unsafe crossing, the participants would receive corresponding text prompts, and then the avatar would return to the starting point and perform the street crossing task again until the end of each task. We did not limit the time it took for participants to cross the street at a time, but in each dual-task, participants were asked to cross the street as much as they could safely. The total duration of the procedure was around 10∼15 min.

**FIGURE 4:**
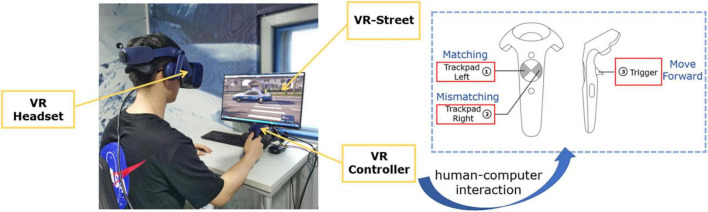
The experimental diagram of the participants and the operation method of the handle.

A diagram of the participants and the operation method of the VR controller is shown in Figure 4. The participant’s walking and stopping were controlled by the VR controller. When the trigger on the handle was pressed, the participant’s virtual avatar moves forward, and when the trigger was released, the avatar stops moving. The avatar walks at a fixed speed of 1.5 m/s in the virtual scene, and the direction is controlled by the HMD, consistent with the direction of the participant’s head. When participants roamed on the virtual street, the visually stimulating content appeared in the center of their field of vision and did not disappear when they turned their heads. The size of Chinese characters and blocks was carefully designed; neither blocked the participants’ field of vision nor was it too small to see clearly. When the participants judged that the visual stimuli matched the auditory stimuli, they were required to touch the left side of the pad of the handle. In case of mismatch, they touched the right side of the pad of the handle. Visual stimuli and auditory stimuli appeared synchronously.

## 4. Data collection

### 4.1. Neuropsychological test

Basic Cognitive Capacity Test is a test for people aged 10-90 (Acta Psychologica Sinica, 2001). In this study, we used its second edition. It can evaluate cognitive abilities in five categories: processing speed, working memory, visuospatial ability, episodic memory and speech ability, with a score range of 0-19. This cognitive test has good reliability, validity and internal consistency and has been verified to accurately evaluate the cognitive ability of different populations.

### 4.2. Subject

**FIGURE 5:**
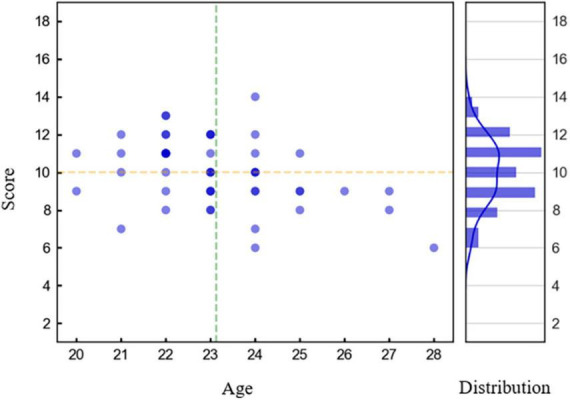
Distribution of basic cognitive capacity test scores on the *y*-axis with respect to age on the *x*-axis. The horizontal line represents the mean score, and the vertical line represents the mean age. The subgraph on the right shows the distribution of subjects in different fractional segments.

The experiment procedures were approved by Guangzhou First People’s Hospital (202002030262). We recruited 50 healthy subjects (M = 23.12, SD = 1.67) aged 20-28 years from college students in Guangzhou to participate in the experiment, including 32 males and 18 females. Their average length of education is 14 years. All subjects received a detailed description of the experiment and signed informed consent before the experiment. The main graph in Figure 5 shows the distribution of subject scores with age, and the subgraph shows the distribution of subject scores.

### 4.3. Procedure

The experimental procedure is shown in Figure 1. The experiment is mainly divided into two stages: practice and data collection. At the beginning, the researchers introduced the VE, the experimental content, the experimental process and the operation method of the equipment to the participants. Then was the practice stage, the researchers helped the participants wear the head mounted display and enter the practice task to make the participants fully familiar with the whole process and human-computer interaction methods. First, the participants had 2 min to walk freely in the virtual scene to become familiar with the scene. Then, the participants were asked to practice Dual-Task I, Dual-Task II and Dual-Task III. The number of stimuli for each dual-task during the practice phase was set to 30. The practice stage was followed by a 2-min rest period, the researchers played a black background picture in the HMD, during which the participants needed to relax and remain calm. Next, participants were asked to complete the data collection stage experiment, including Dual-Task I, Dual-Task II and Dual-Task III, and the number of stimuli per dual-task was 72. The Stroop task performance data and behavior data were recorded during the experiment. On average, the whole procedure took approximately 30 min.

## 5. Data analysis

### 5.1. Feature extraction

We introduced a series of features extracted from participants’ Stroop task performance and behavior performance in the virtual reality task state and established a supervised machine learning model based on these data.

For Stroop task performance, the correct number of answers (CNST) and response time (RTST) were recorded.

For the behavior data, first, the number of safe crossings (NSC), number of unsafe crossings (NUC) and number of attempts (NA) were measured, where NA was the sum of NUC and NSC. Then, we recorded some time indicators, including time spent crossing the street (TSC) and time to collision (TTC). TSC represents the total time taken by the participants to cross the street, starting from when the virtual avatar stood at the starting point until he reached the destination marked by the arrow. Additionally, the maximum and minimum values of TSC in each dual-task were considered. TTC was the safe time interval for crossing the street. When the virtual avatar passed through point A on the road, the recording time was T_1_. When the vehicle reached point A after the avatar passed, the recording time was T_2_, and TTC was defined as:


(1) $$T\,T\,C=T_{2}-T_{1}$$

Meanwhile, we measured the trajectory and velocity of participants while crossing the street in the virtual environment. The trajectory was the sample values of a sequence of coordinates of the participant’s path, which was defined as (*x*_*i*_,*y*_*i*_),*i*=1,2⋯*N*, the representation of *x*-axis and *y*-axis in VE is shown in Figure 2. To measure the ability of participants to maintain direction when crossing the street, the standard deviation (SDX) and entropy (ETPX) of the trajectory in the x-axis direction were extracted. SDX was the standard deviation of the trajectory and was used to measure the dispersion degree of the participant’s *x*-axis trajectory. SDX was computed by equation (2). ETPX was the entropy of the trajectory in the *x*-axis direction, which was defined by Equation (3),


(2) $$S\,D\,X=\sqrt{\frac{1}{N-1}\,\sum_{i=1}^{N}{\left(x_{i}-\bar{x}\right)}^{2}}$$

(3) $$E\,T\,P\,X=-\sum_{i=1}^{N}P\,\left(x_{i}\right)\,\log_{2}\left(P\,\left(x_{i}\right)\right)$$

Velocity was recorded in the x and y directions, and the mean velocity (MV) and standard deviation of velocity (SDV) were extracted. MV and SDV were computed by Equations (4) and (5), respectively;


(4) $$M\,V=\frac{1}{N}\,\sum_{i=1}^{N}v_{i}$$

(5) $$S\,D\,V=\sqrt{\frac{1}{N-1}\,\sum_{i=1}^{N}{\left(v_{i}-M\,V\right)}^{2}}$$

smoother movements incur a lower SDV (Kotsavasiloglou et al., 2017). Furthermore, the number of head turns (HT) and the F-norm of the attention matrix (FN) before crossing the street were measured by the Euler angle of HMD. The attention matrix was recorded once in each frame, and its size was 180×360. FN was used to measure the degree of distraction of participants’ attention, and it was computed as:


(6) $$F\,N=\sqrt{\sum_{i=1}^{180}\sum_{j=1}^{360}{\left|a_{i\,j}\right|}^{2}}$$

where *a*_*ij*_ was the element in the attention matrix, and a larger FN represented more distracted attention. HT refers to the number of head turns before crossing the street. The rotation angle of the participant’s head was defined as θ, which was the included angle between the participant’s orientation and the negative direction of the y-axis. When the head of the participant rotated more than 30° to either side (θ < 30°), it was recorded as a head rotation.

A total of 68 dimensional features were extracted. For each type of feature, its values in three dual-tasks were counted, and the average of the three values was counted as the overall performance of the participants. A detailed description of all feature sets is shown in Table 1. To select a specific feature subset highly related to the processing speed level, the RFE algorithm (Guyon et al., 2002) was applied to feature selection. The RFE algorithm starts from all features and assigns a weight to each feature, and the feature with the minimum weight is excluded from the feature set. This loop recurses until the number of remaining features reaches the required number of features. Finally, the top 10 features were selected as the optimal feature subset.

**TABLE 1:** Definition and description of features.

Feature category	Name	Description
Stroop performance	CNST	Correct number of answers
	RTST	Response time
Behavior data	NUC	Number of unsafe crossings
	NSC	Number of safe crossing
	NA	Number of attempts to cross the street
	TSC	Time spent crossing the street
	TSC_MAX	Maximum time spent crossing the street
	TSC_MIN	Minimum time spent crossing the street
	TTC	Time to collision
	SDX	Standard deviation of the trajectory in *x*-axis
	ETPX	Entropy of the trajectory in *x*-axis
	MV_*X*,*Y*_	Average velocity in *x*-axis and *y*-axis
	SDV_*X*,*Y*_	Standard deviation of velocity in *x*-axis and *y*-axis
	FN	F-norm of attention matrix
	HT	Head turns

### 5.2. Statistical analysis

The statistical analysis method used the Pearson correlation coefficient to find the relationship between the dual-task features and the cognitive test results to initially explore the validity of the extracted features. A p-value less than 0.05 was determined to be a statistically significant difference.

### 5.3. Machine learning model

The LASSO, SVR and XGBoost (eXtreme Gradient Boosting) algorithms were used to establish regression models to estimate the cognitive processing speed scores. These three regression models come from different learner categories. Lasso regression (Tibshirani, 2011) is a linear regression model that uses L1 regularization to limit feature weights. Support vector regression (SVR) (Vapnik, 1998) is a regression method based on support vector machine (SVM). XGBoost (Chen and Guestrin, 2016) is an integrated machine learning algorithm based on decision tree. Comparing the results of different models in the study can evaluate the performance of the model more comprehensively, and finally select the best model to achieve the best prediction effect.

Firstly, the Stroop task performance and behavior data were taken as the input of the model, and the performance of the estimated processing speed score of the regression model was analyzed. Secondly, the performance of different types of features in estimating cognitive scores was analyzed. Finally, we compared the results of using each dual-task separately. The optimal value of the regular term coefficient alpha of LASSO and parameter C of SVR were selected through cross validation within the training set. The regression performance of each model was calculated using LOSO-CV. That is, a total of 50 iterations of cross validation were performed, in which the data obtained from 49 participants were used to train an estimation model and the data belonging to the left-out participant were used to evaluate the trained model. The average value of 50 iteration results was used to evaluate the performance of the model.

The evaluation indices of model performance were mean absolute error (MAE), relative accuracy (ACC) and correlation coefficient (CC). The calculation formula of MAE is as follows:


(7) $$M\,A\,E=\frac{1}{n}\,\sum_{i=1}^{n}\left|{\hat{y}}_{i}-y_{i}\right|$$

where *n* is the number of participants, *y* is the true value and $\hat{y}$ is the predicted value. The smaller the value of MAE, the better the prediction performance of the model.

ACC reflects the relative error between the true value and the predicted value, with a range of 0-1. The closer it is to 1, the better the performance of the model. The formula is as follows:


(8) $$A\,C\,C=1-\frac{1}{n}\,\sum_{i=0}^{n}\frac{\left|\left(y_{i}-{\hat{y}}_{i}\right)\right|}{y_{i}}$$

CC reflects the linear relationship between the true value and the predicted value, which ranges from 0 to 1. The formula is as follows:


(9) $$C\,C=\frac{c\,o\,v\,\left(y,\hat{y}\right)}{\sigma_{y}\,\sigma_{\hat{y}}}$$

where σ_*y*_ and $\sigma_{\hat{y}}$ represent the standard deviation of *y* and $\hat{y}$, and $c\,o\,v\,\left(y,\hat{y}\right)$ represents the covariance of *y* and $\hat{y}$. The greater the CC, the greater the correlation between the predicted value and the true value, and the better the prediction performance of the model.

## 6. Results

### 6.1. Statistical analysis

To understand the behavior and cognitive performance in dual-tasks, various Stroop task performance features and behavioral features were extracted. Pearson’s correlation coefficient was used to find the relation between features and cognitive test results, as shown in Table 2. It is revealed that there were 28 features that had a certain degree of correlation with the target variables (|*r*|>0.3,*p*<0.05). Among them, all Stroop tasks showed a significant correlation, indicating that VR Stroop in dual-tasks was effective for evaluating processing speed. In the task of street-crossing, NSC in Dual-Task I and Dual-Task II showed strong correlation with the cognitive test results, while HT, TSC and TSC_MIN showed medium correlation. The results indicated that these features may provide some useful information in the further evaluation.

**TABLE 2:** Correlation analysis of features and cognitive test results.

Feature	Correlation	*p*-value
RTST in Dual-Task I	-0.774	**
RTST in Dual-Task II	-0.705	**
RTST in Dual-Task III	-0.728	**
Average RTST in three dual-tasks	-0.777	**
CNST in Dual-Task I	0.709	**
CNST in Dual-Task II	0.669	**
CNST in Dual-Task III	0.683	**
Average CNST in three dual-tasks	0.718	**
NSC in Dual-Task I	0.524	**
NSC in Dual-Task II	0.528	**
NSC in Dual-Task III	0.343	*
Average NSC in three dual-tasks	0.573	**
NUC in Dual-Task I	-0.337	*
NUC in Dual-Task II	-0.102	0.481
NUC in Dual-Task III	-0.019	0.896
Average NUC in three dual-tasks	-0.215	0.135
NA in Dual-Task I	0.165	0.254
NA in Dual-Task II	0.387	**
NA in Dual-Task III	0.287	*
Average NA in three dual-tasks	0.315	*
TTC in Dual-Task I	0.102	0.483
TTC in Dual-Task II	-0.039	0.788
TTC in Dual-Task III	-0.019	0.898
Average TTC in three dual-tasks	0.040	0.784
TSC in Dual-Task I	-0.317	*
TSC in Dual-Task II	-0.355	*
TSC in Dual-Task III	0.009	0.954
Average TSC in three dual-tasks	-0.310	*
TSC_MAX in Dual-Task I	-0.190	0.187
TSC_MAX in Dual-Task II	-0.240	0.093
TSC_MAX in Dual-Task III	-0.025	0.863
Average TSC_MAX in three dual-tasks	-0.271	0.057
TSC_MIN in Dual-Task I	-0.365	**
TSC_MIN in Dual-Task II	-0.365	**
TSC_MIN in Dual-Task III	0.063	0.663
Average TSC_MIN in three dual-tasks	-0.022	0.881
HT in Dual-Task I	0.341	*
HT in Dual-Task II	0.455	**
HT in Dual-Task III	0.458	**
Average HT in three dual-tasks	0.438	**
FN in Dual-Task I	-0.067	0.644
FN in Dual-Task II	-0.248	0.083
FN in Dual-Task III	-0.198	0.168
Average FN in three dual-tasks	-0.208	0.148
SDX in Dual-Task I	0.159	0.269
SDX in Dual-Task II	0.091	0.529
SDX in Dual-Task III	0.122	0.398
Average SDX in three dual-tasks	0.131	0.364
ETPX in Dual-Task I	0.155	0.282
ETPX in Dual-Task II	-0.145	0.314
ETPX in Dual-Task III	-0.163	0.257
Average ETPX in three dual-tasks	-0.072	0.622
MVX in Dual-Task I	0.174	0.227
MVX in Dual-Task II	0.264	0.064
MVX in Dual-Task III	0.251	0.079
Average MVX in three dual-tasks	0.247	0.083
MVY in Dual-Task I	0.321	*
MVY in Dual-Task II	0.461	**
MVY in Dual-Task III	0.314	*
Average MVY in three dual-tasks	0.402	**
SDVX in Dual-Task I	0.114	0.431
SDVX in Dual-Task II	0.165	0.252
SDVX in Dual-Task III	0.025	0.866
Average SDVX in three dual-tasks	0.112	0.438
SDVY in Dual-Task I	0.036	0.804
SDVY in Dual-Task II	0.085	0.557
SDVY in Dual-Task III	-0.044	0.763
Average SDVY in three dual-tasks	0.026	0.856

*p 0.05; **p 0.01.

### 6.2. Regression model on cognitive processing speed

**FIGURE 6:**
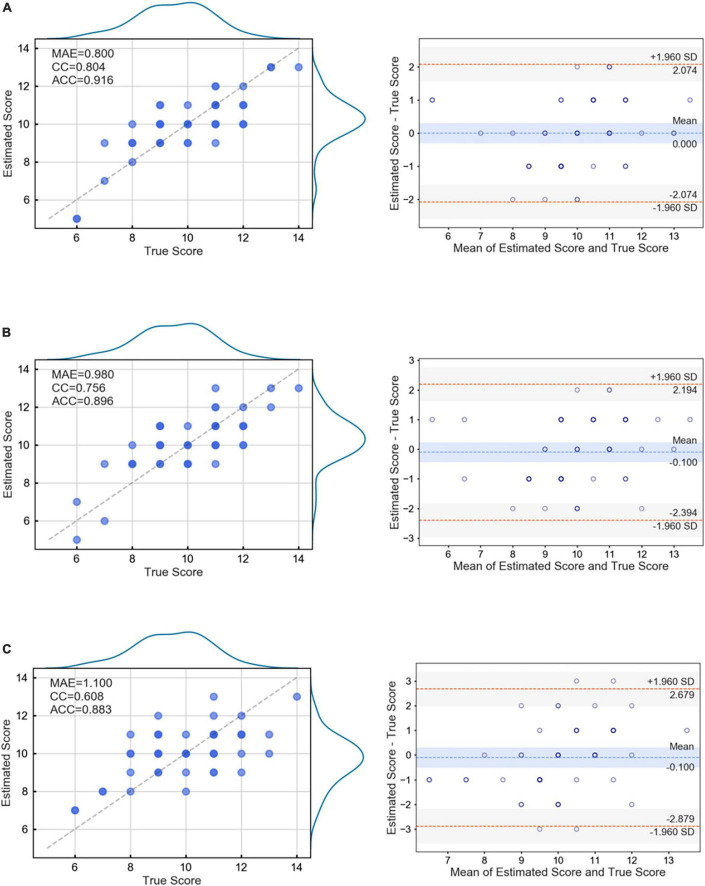
Fitting result and Bland–Altman plot of the three regression models. **(A)** LASSO, **(B)** SVR, **(C)** XGBoost.

The estimation performance of three different machine learning regression models (LASSO, SVR, and XGBoost) was compared. Figure 6 shows the scatter plot and Bland–Altman plot between the estimated scores and true scores. LASSO regression obtained the best results (MAE = 0.800, ACC = 0.916, CC = 0.804). In the Bland – Altman diagram, all prediction points of LASSO and SVR fell within the 95% confidence interval, indicating that the difference between the prediction results and the actual results was small, and there was no significant systematic deviation. The preliminary prediction results were relatively reliable. However, owing to the small sample size, it was still necessary to expand the sample size for further evaluation in the future. The presented results demonstrated that the participants’ performance in VR-Street could accurately estimate the score of the clinical scale.

In each iteration of LOSO-CV, the RFE feature selection algorithm was applied to select feature subsets containing 10 features in the training set, and a total of 50 feature subsets were selected. All selected features were counted and sorted according to the number of times they were selected. Table 3 lists the 10 features selected more than 25 times in the LASSO regressor. The absolute value of correlation coefficient between most features in the table and cognitive test results was greater than 0.3, including RTST in Dual-Task I, TTC in Dual-Task II, TSC_ MIN in Dual-Task III, HT in Dual-Task II, etc. Among the Stroop task performance features, RTST in Dual-Task I and Dual-Task III and CNST in Dual-Task III were selected. All other features are behavioral features. These findings prove that the combined features of dual-tasks have an important contribution to the prediction results, and it is reasonable to use the feature subset to estimate the processing speed score.

**TABLE 3:** The 10 most important features for evaluating processing speed.

Features	Count (Prec.)
RTST in Dual-Task I	50 (100%)
TTC in Dual-Task II	50 (100%)
TSC_MIN in Dual-Task III	50 (100%)
HT in Dual-Task II	50 (100%)
NSC in Dual-Task II	49 (98%)
SDVX in Dual-Task III	49 (98%)
Average value of TSC_MIN in three dual-tasks	48 (96%)
CNST in Dual-Task III	46 (92%)
RTST in Dual-Task III	43 (86%)
Average value of SDX in three dual-tasks	40 (80%)

### 6.3. Regression model on different types of features

To investigate the impact of different data on the evaluation, models were established using Stroop task performance data, behavior data and combined data, and the results were analyzed. A feature selection algorithm was not applied to ensure fairer comparison results between different feature subsets. Since LOSO-CV was applied, we discussed the average MAE and ACC of all individuals.

**FIGURE 7:**
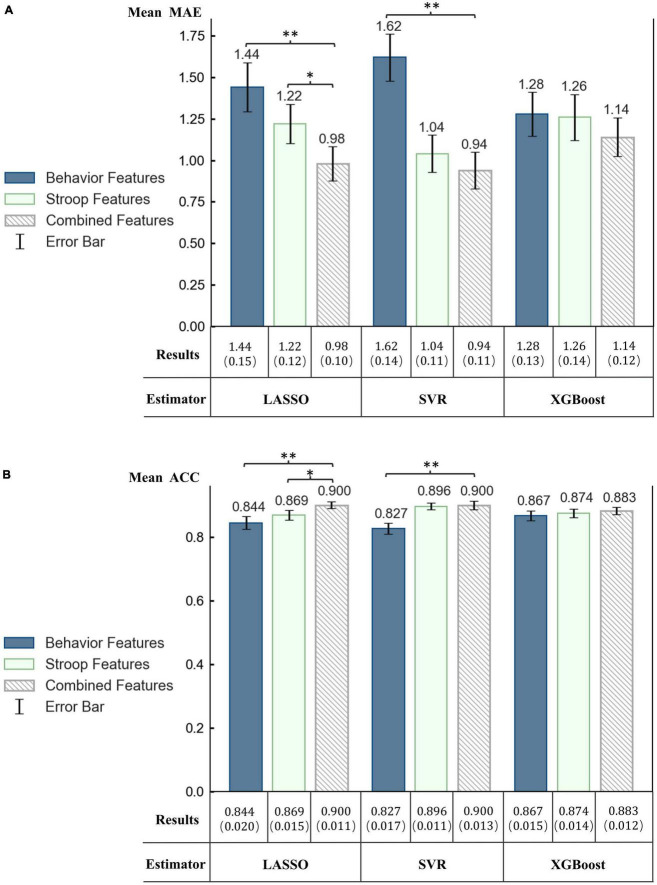
Performance of regression models using different feature combinations. **(A)** Mean MAE and the standard error of the mean (SEM) in parentheses. **(B)** Mean ACC and SEM in parentheses. *p 0.05. ^**^p 0.01.

Figure 7 shows the performance (MAE and ACC) for different combinations of the feature set. The three regressors showed similar trends, and on average, the combined feature set of dual-task achieved the best results. Through paired t-test analysis, it was found that in the LASSO model, the estimated results of the combined features were significantly better than the results of the Stroop task performance (t-stats = −2.064, with *p* = 0.044) and the results of the behavior data (t-stats = −3.031, with *p* = 0.004). In addition, the results of combined features in SVR were also significantly better than those of behavioral features (t-stats = −4.462, with *p* = 4.742e-05). The accuracy achieved by the combination of features indicated that dual-tasks can more fully reflect the individual’s processing speed level.

### 6.4. Regression model on one of the three dual-tasks

We trained a model separately for each dual-task and compare the estimation results obtained by the three models. The aim was to investigate whether the three tasks in the Stroop test have any difference in the evaluation results in VR-Street. The MAE of the results of each mode was calculated to measure the performance of the regression models.

**FIGURE 8:**
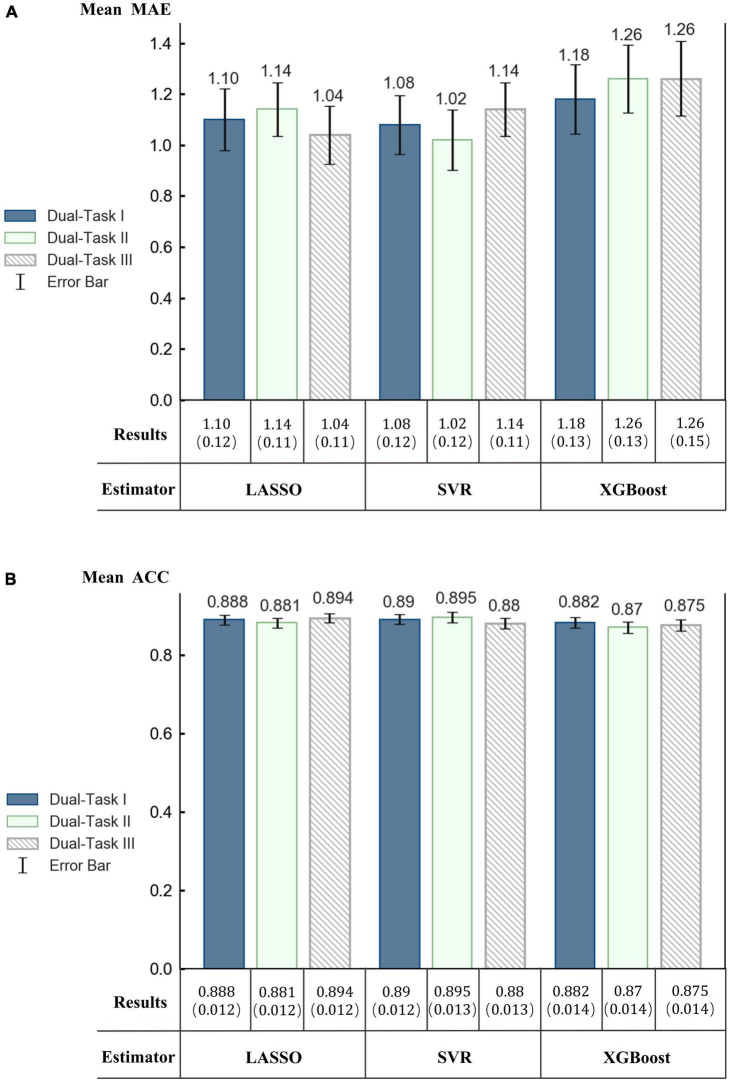
Performance of regression models using three dual-tasks separately. **(A)** Mean MAE and the standard error of the mean (SEM) in parentheses. **(B)** Mean ACC and SEM in parentheses.

Comparisons of MAE are shown in Figure 8. The results showed that there is no significant difference between the prediction results of the three dual-tasks, and on average, the combined model prediction results of multiple dual-tasks have better prediction performance, but when LASSO or SVR models are used, the prediction results of a single dual-task are also acceptable.

## 7. Discussion and conclusion

In this study, we introduced a method based on virtual reality and machine learning to estimate cognitive processing speed scores. To this end, we developed VR-Street, simulated the virtual street-crossing task and embedded the Stroop test as a distraction task. The VR-Street was applied to 50 healthy adult participants, from which Stroop task performance and behavior performance were collected. In order to estimate the cognitive processing speed score, we developed a machine learning method, extracted 68 dimensional features. Firstly, we used Pearson correlation to find the relation between features and cognitive tests. All the Stroop task features and some behavioral features had a certain degree of correlation with cognitive test scores. Participants with better processing speed showed more frequent head turns, however, in the separate street-crossing task, the participants’ performance was opposite (Stratton et al., 2017). This result may be due to the fact that in the dual-task condition, people with poor cognitive processing speed have more difficulty processing two tasks at once, so they may pay more attention to their attentional resources. This will cause them to reduce the frequency of head turns to conserve attentional resources, thereby performing better in the Stroop task. Then, we used LOSO-CV to verify the accuracy of the evaluation. The results showed that our method can accurately estimate the processing speed score of clinical validation (MAE = 0.800, ACC = 0.916, CC = 0.804). Most previous studies only analyzed part of the behavior data in VR-Street, such as the number of head turns and the time spent crossing the street (Neider et al., 2011; Banducci et al., 2016; Stratton et al., 2017). On this basis, this study added the data features of trajectory and speed, and considered the performance of distracted tasks. Through ranking the contribution of feature subsets filtered by RFE feature selection algorithm, it was found that the performance of Stroop tasks showed an important contribution, especially the response time, which was an important manifestation of processing speed (Lu et al., 2017). The standard deviation of the trajectory and velocity on the *x*-axis also has a high ranking, which indicates that the trajectory and speed features in VR tasks may have potential in cognitive evaluation (Tsai et al., 2021). Additionally, The three machine learning models all showed a similar trend that the estimation result of the combination of behavior performance and distraction task performance was more accurate than that of single type of data. And the results of LASSO model showed significant differences. Due to the interaction between the street-crossing task and the Stroop task, which increases the cognitive load of participants when they complete the two tasks at the same time. Under high load conditions, participants need to better manage processing speed and attention, so as to effectively process information and make decisions in a short time (Lavie, 2005). Therefore, the design of dual-tasks in this study better mobilized participants’ attention and cognitive resources, promoted their conversion and coordination between different tasks, and thus more comprehensively reflected participants’ cognitive processing speed. This provided a reference for more future studies to apply multi task data to cognitive assessment. Finally, the evaluation results of the three dual-tasks were compared. The results showed that, on average, the estimation results of the combination of dual-tasks were better than their respective results. When LASSO or SVR model is used, the estimation result of a single dual-task is acceptable. Although the Stroop tasks in the three dual-tasks involved different cognitive processes, there was no significant difference between the prediction results, indicating that the three Stroop sub-tests contributed similarly to the prediction of processing speed. A more comprehensive assessment of an individual’s cognitive abilities can be achieved through a combination of different dual-tasks. We will further study the application of these three dual-tasks in VR-Street in future work, and explore the feasibility of using a single dual-task for evaluation.

VR-Street was a preliminary attempt. Compared with other studies, we embedded Stroop test in VR-Street as a distraction task and tried to establish a machine learning model for quantifying the evaluation results. We imagined that users can use VR-Street for self-management, and quantitative cognitive scoring provides them with the opportunity to self monitor the track of processing speed level changes. The score can also be reported to the clinician, so as to develop a more targeted care plan. When VR-Street is applied to populations with different pathologies, the display size and volume of the Stroop stimulus can be adjusted. Specifically, for patients with visual-spatial impairments, environmental sounds such as the engine noise of vehicles can be increased to aid their identification of approaching vehicles. In general, our work has great application potential; It can not only provide reference for the quantification of behavior in virtual environment, but also provide reference and help for the clinical application of cognitive function evaluation methods based on virtual reality.

## 8. Limitations and future work

Specifically, our research has limitations and necessitates further work in the future. First, the number of participants in this experiment was limited, and they were healthy college students, which may hinder the universality of its application to the general population. Expanding the data set to deal with the diagnosis of a wider age group and patients with cognitive impairment is one of our future work. At the same time, machine learning models with stronger generalization ability will be developed. When the system is applied to individuals with different ages or pathological conditions, the difficulty of the task can be adjusted by changing the total number of stimuli in the Stroop task, the display time of Stroop stimuli, the distance between vehicles, and the speed of vehicles to adapt to the user’s cognitive level. Machine learning models with stronger generalization ability can ensure accurate evaluation under different system settings. Second, as this is a preliminary study, we use the operation of the handle to control the movement of the avatar in the virtual scene, which may limit the immersion of the system. In the future research, we plan to use omnidirectional platform instead of handle to provide the means to walk in the virtual scene, and further explore the evaluation method of processing speed under cognitive-motor dual-task. Third, the measurement of physiological data, such as EEG or eye movement tracking, can be added to our work to explore the application of physiological data in cognitive assessment in virtual environments. Finally, the assessment of more cognitive functions should be explored. Our work only focuses on cognitive processing speed at present, which limits the wider screening of cognitive function. Therefore, our future work will explore the feasibility of using VR-Street to evaluate different cognitive functions, and apply it to the study of cognitive decline associated with depression. We will also further explore the application of this system in cognitive training.

## Data availability statement

The raw data supporting the conclusions of this article will be made available by the authors, without undue reservation.

## Ethics statement

The studies involving human participants were reviewed and approved by Guangzhou First People’s Hospital, The Second Affiliated Hospital, South China University of Technology, Guangzhou, China. The patients/participants provided their written informed consent to participate in this study.

## Author contributions

YZhou was responsible for the entire study. YZhou, ZY, LS, XX, and LZ contributed to the experiment design. YZhou, ZX, and YZhao contributed to the VR system design. LZ contributed to the screening of subjects. ZY and XT helped with the data collection. YZhou, ZY, LS, XX, and LZ were responsible for the data analysis. All authors listed have made a direct and intellectual contribution to the work.
